# A Case Report and Surgical Video Presentation on the Anterior Approach for Right Anterior Hepatectomy: A Solution to Complex Biliary Pathologies

**DOI:** 10.7759/cureus.83251

**Published:** 2025-04-30

**Authors:** Camila Sotomayor, Cristobal Vildosola, Pablo Achurra, Patricia Rebolledo, Eduardo Viñuela, Nicolás Jarufe, Jorge Martínez, Eduardo Briceño

**Affiliations:** 1 Department of Hepatobiliary and Pancreatic Surgery, Pontificia Universidad Católica de Chile, Santiago, CHL

**Keywords:** anterior approach, hepatolithiasis, laparoscopic hepatectomy, minimally invasive techniques, right anterior hepatectomy

## Abstract

Intrahepatic hepatolithiasis, characterized by the presence of stones in the intrahepatic bile ducts, is often associated with biliary strictures and chronic inflammation, complicating surgical management. Patients with this condition frequently experience recurrent cholangitis and face a high risk of hepatic complications. Segmental laparoscopic hepatectomy is an effective therapeutic option that removes both stones and the affected liver tissue.

This study presents the case of a 40-year-old female with a history of laparoscopic cholecystectomy three years ago, a lesion in the right hepatic duct, a user of a bile duct stent, and under follow-up with hepato-pancreato-biliary (HPB) surgery. She was currently asymptomatic. Magnetic resonance cholangiopancreatography (MRCP) revealed stenosis of the anterior duct, suggestive of hepatolithiasis. The patient was admitted for elective surgical resolution and treated with right anterior hepatectomy using an anterior approach under conditions of significant inflammation.

This analysis was based on a retrospective review of the patient’s medical records and intraoperative video documentation.

The procedure was performed at Hospital Clínico Pontificia Universidad Católica de Chile. An anterior approach was chosen to perform the anterior right segmental hepatectomy due to the severe inflammation, which made identifying critical anatomical structures challenging. The surgery was performed laparoscopically to minimize surgical trauma and promote faster recovery. Intraoperative rapid biopsies were used to ensure clear margins.

The surgery successfully resected the affected hepatic segment, eliminating both the stones and the stricture. The patient had an uneventful postoperative course. No re-interventions were required, and the patient was discharged in good general condition, with no recurrence observed during the initial follow-up.

This case report was previously presented as a meeting video at the 2025 AHPBA meeting held in Miami, USA, on March 20-23, 2025.

## Introduction

Hepatolithiasis is defined as the presence of gallstones within the intrahepatic bile ducts proximal to the confluence of the right and left hepatic ducts, which can lead to complications such as recurrent cholangitis, biliary strictures, segmental hepatic atrophy, and cholangiocarcinoma (CCA) [1]. Although it is more prevalent in East Asia, its incidence in Western countries has been rising due to migration, resulting in an increased global incidence overall [2].

Management depends on the symptoms, extent and location of the stones, and hepatic or biliary involvement. In cases associated with biliary strictures, segmental atrophy, or failed endoscopic interventions, hepatectomy is often indicated. The most important goals during treatment of hepatolithiasis are removal of stones, resolution of strictures, and prevention of cholangitis to hinder the progression of the disease and development of CCA [2].

Surgical treatment plays a central role in the management of hepatolithiasis, particularly in patients with localized disease, recurrent cholangitis, biliary strictures, or segmental hepatic atrophy [2,3]. Hepatectomy not only reduces the risk of stone recurrence but also lowers progression to CCA [4,5].

Minimally invasive techniques have gained prominence in the surgical management of hepatolithiasis. According to a systematic review and meta-analysis by Li et al., laparoscopic hepatectomy offers several advantages over open surgery. These include significantly reduced intraoperative blood loss, shorter hospital stay, lower postoperative complication rates, and faster recovery times, without compromising stone clearance or long-term outcomes. Importantly, the laparoscopic approach was found to be equally effective in achieving complete removal of calculi and in reducing recurrence while minimizing surgical trauma [6].

Despite being considered a rare disease in Western countries, especially in South America, a significant number of cases have been reported since the early 1960s. Recent case series regarding the treatment of this challenging disease, mostly from Brazil and Chile, have shown an apparent increase in the incidence of hepatolithiasis in both countries [7]. In a multicenter study involving 149 patients (72 from Chile and 77 from Brazil), it was shown that most cases involved symptomatic disease, often requiring surgical intervention due to complications such as biliary strictures, cholangitis, or segmental liver atrophy. The majority of patients had unilobar disease (95.9%), and 83.2% were symptomatic. Liver resection was performed in all cases, achieving 100% immediate stone clearance and a low recurrence rate (5.4%). These findings reinforce hepatectomy as an effective and definitive treatment for patients with unilobar disease or irreversible biliary damage, offering excellent long-term outcomes despite its higher initial morbidity (30.9%) and complexity [8].

The anterior approach to liver resection consists of performing parenchymal transection before liver mobilization, thereby limiting manipulation and lowering the risk of bile duct injury in cases with inflammation or fibrosis. Right anterior sectionectomy, also referred to as right anterior hepatectomy, has become an important surgical strategy for treating complex biliary conditions affecting the anterior liver segments (V and VIII).

In this report, we present a case of complex hepatolithiasis managed successfully with an anterior approach right anterior hepatectomy, emphasizing the utility of this surgical strategy in addressing difficult biliary tract disease.

## Case presentation

We report the case of a 40-year-old woman with a history of laparoscopic cholecystectomy performed three years earlier, complicated by iatrogenic injury to the right hepatic duct. The patient subsequently developed recurrent hepatolithiasis, requiring multiple endoscopic retrograde cholangiopancreatography (ERCP) procedures and placement of an indwelling biliary stent. At present, she remains asymptomatic.

**Figure 1:**
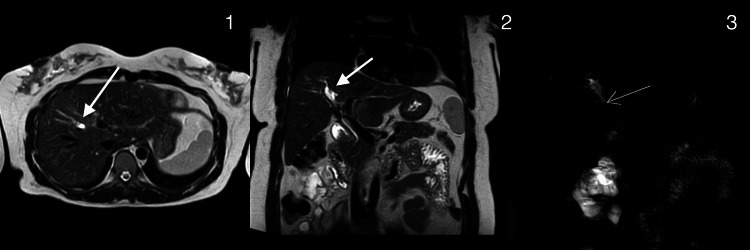
Preoperative magnetic resonance cholangiopancreatography (MRCP) 1. Axial plane. Arrow: Anterior segmental intrahepatic biliary ductal dilation 2. Coronal plane. Arrow: Anterior segmental intrahepatic biliary ductal dilation 3. Reconstructed biliary phase on MRCP. Arrow: Scant heterogeneous material within the lumen of the segmental anterior intrahepatic bile duct, consistent with hepatolithiasis

During routine follow-up by the hepatobiliary surgery team, MRCP revealed stenosis of the anterior sectoral bile duct with findings suggestive of hepatolithiasis (Figure 1).

As part of the preoperative workup, laboratory tests were completed (Table 1).

**Table 1:** Laboratory tests CRP, C-reactive protein; Hb, hemoglobin; Hct, hematocrit; AST (SGOT), aspartate aminotransferase; ALT (SGPT), alanine aminotransferase; GGT, gamma-glutamyl transferase; ALP, alkaline phosphatase; total bilirubin, total serum bilirubin; direct bilirubin, conjugated bilirubin; INR, international normalized ratio; creatinine, serum creatinine; estimated GFR (CKD-EPI), estimated glomerular filtration rate (Chronic Kidney Disease Epidemiology Collaboration equation); CA 19-9, carbohydrate antigen 19-9; platelet count, platelet concentration per microliter; WBC, white blood cell count; RBC, red blood cell count; MCV, mean corpuscular volume; MCH, mean corpuscular hemoglobin; RDW, red cell distribution width; ESR, erythrocyte sedimentation rate.

TEST	Results	Reference range
C-reactive protein (CRP) mg/dL	0.46 mg/dL	Less than 0.5
Hemoglobin g/dL	10.7 g/dL	12.0–16.0
Hematocrit %	31.9%	36.0–46.0
SGOT (AST) U/L	32 U/L	Up to 35
SGPT (ALT) U/L	28 U/L	Up to 35
GGT U/L	85 U/L	Up to 40
Alkaline phosphatase U/L	110 U/L	30–100
Total bilirubin mg/dL	0.53 mg/dL	Up to 1.0
Direct bilirubin mg/dL	0.26 mg/dL	Up to 0.3
INR	1.0	0.8–1.2
Creatinine mg/dL	0.50 mg/dL	0.50–0.90
Estimated GFR (CKD-EPI)	121 mL/min/1.73 m2	≥ 90
CA 19-9 U/mL	5.9 U/mL	Up to 34.0
Platelet count x10^3/µL	390 x10^3/µL	140–400
WBC count x10^3/µL	8.0 x10^3/µL	4.5–11.0
RBC count x10^6/µL	4.26 x10^6/µL	4.00–5.20
MCV fL	74.9 fL	80.0–100.0
MCH pg	25.1 pg	26.0–34.0
RDW %	16.7%	11.6–14.6
ESR mm/hr	6 mm/hr	1–24

As a result, a laparoscopic right anterior sectionectomy was planned. Intraoperatively, marked inflammation of the surrounding structures secondary to chronic hepatolithiasis was noted, leading to significant distortion of the biliary and vascular anatomy. Given these findings, an anterior surgical approach was utilized (Video 1).

**Video 1:** Anterior approach for right anterior hepatectomy

The surgery began with the mobilization of the right hepatic lobe. Dissection of the pedicle was laborious due to adhesions secondary to a previous cholecystectomy. An intraoperative US was performed, and it was decided to proceed with the anterior approach following the axis of the middle hepatic vein and the right hepatic vein. The Pringle maneuver was applied. Biliary structures to segments V and VIII were identified, dissected, and divided between hem-o-lock staples. Additionally, during the parenchymal transection, all the small veins were coagulated with the harmonic scalpel. Structures of segments V and VIII were identified, and dissection of the elements of the anterior pedicle was carried out. The anterior pedicle was divided with staples. The right hepatic vein was identified, and it was decided to follow this access to continue the hepatic transection. The access of the fissure of Gans was then identified, and the hepatic parenchyma was transected above this. The anterior hepatectomy was completed, and the specimen was extracted using an endocatch bag. Hemostasis was achieved using bipolar coagulation, and hemostatic material was left in place.

The patient's postoperative course was uneventful, and she was discharged in stable condition.

**Figure 2:**
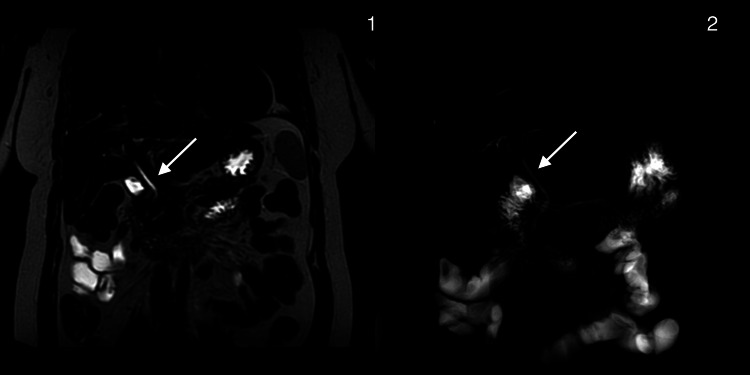
Postoperative magnetic resonance cholangiopancreatography (MRCP) Postsurgical changes consistent with anterior hepatic segmentectomy; no evidence of biliary lithiasis or new areas of intrahepatic biliary dilation 1. Coronal plane. Arrow: The extrahepatic bile duct measures 3 mm in internal diameter at the level of the common hepatic duct. No hypointense filling defects suggestive of intraluminal stones are identified. 2. Reconstructed biliary phase on MRCP. Arrow: The extrahepatic bile duct measures 3 mm in internal diameter at the level of the common hepatic duct. No hypointense filling defects suggestive of intraluminal stones are identified.

In Video 1, we can see the surgery completed with the right hepatic vein preserved and intact. Drains were placed. Postoperative control with MRCP showed a resolution of hepatolithiasis and a fine bile duct (Figure 2).

## Discussion

There is an association between a history of previous cholecystectomy and right liver lobe hepatolithiasis due to iatrogenic vascular injuries during cholecystectomy [1,9]; however, this requires further studies. The present case presented with a history of cholecystectomy and right liver lobe hepatolithiasis.

There are several treatment options available for intrahepatic stones, including endoscopic and percutaneous treatments, with or without lithotripsy, using choledochoscopy, and surgical interventions such as hepatectomy or biliary bypass. The main goals in managing hepatolithiasis are to extract all intra- and extrahepatic stones and to remove any bile duct stenosis, affected bile duct drainage areas, and atrophic segments. It is important to note that the chronic inflammatory process caused by stones in the intrahepatic bile ducts is a recognized risk factor for cholangiocarcinoma, occurring in up to 21.2% of cases [2,9].

The laparoscopic approach for hepatectomy in hepatolithiasis has gained acceptance due to its minimally invasive nature, which has proven to be a safe and effective technique, with lower estimated blood loss during surgery, fewer postoperative complications, reduced length of hospital stay, and faster recovery of intestinal function compared to conventional approaches [6].

Hepatectomy has been reported to have excellent results, including the removal of stones and reducing the risk of recurrence of stones. Liver resection can reduce the risk of CCA by removing the presence of irreversible lesions such as parenchymal atrophy, biliary stenosis, or severe fibrosis of the affected segment/lobe [2,8,9].

Liver resection demonstrates significantly lower recurrence rates of calculi compared with other techniques. This varies between 5.8% to 9.4%, and non-surgical approaches like ERCP and percutaneous lithotripsy are associated with substantially higher recurrence rates, ranging from 20% to 60% in different studies [8].

The anterior approach to hepatectomy involves initial vascular inflow control, completion of parenchymal transection, and complete venous outflow control before the right liver is mobilized, minimizing manipulation and reducing bile duct injury in inflamed or fibrotic tissue. Right anterior sectionectomy, or right anterior hepatectomy, has emerged as a valuable surgical approach for managing complex biliary pathologies involving the anterior segments (V and VIII) of the liver. This could lead us to better surgical outcomes, such as lower recurrence rates of calculi or postoperative complications like biliary fistula, bile leakage, or intra-abdominal abscess.

## Conclusions

Laparoscopic right anterior hepatectomy represents a safe and effective treatment strategy for complex biliary conditions, particularly in the presence of intrahepatic lithiasis, strictures, and severe fibrosis. The anterior approach facilitates parenchymal transection in an inflamed and distorted surgical field, minimizing the risk of injury to vital structures. In this case, complete resection of the affected segments enabled definitive management, with no early recurrence and an uneventful postoperative recovery.

This case underscores the utility of minimally invasive liver resection in selected patients where conventional or non-surgical therapies are limited by anatomical or inflammatory challenges. When endoscopic or percutaneous interventions fail or are not feasible, liver resection remains a definitive option with favorable long-term outcomes, reducing the risk of recurrent disease and malignant transformation.
